# Allelic and genotypic frequencies in polymorphic Booroola fecundity gene and their association with multiple birth and postnatal growth in Chhotanagpuri sheep

**DOI:** 10.14202/vetworld.2016.1294-1299

**Published:** 2016-11-25

**Authors:** Thanesh Oraon, D. K. Singh, Mayukh Ghosh, S. S. Kullu, Rajesh Kumar, L. B. Singh

**Affiliations:** 1 Department of Animal Breeding and Genetics, Ranchi Veterinary College, Birsa Agricultural University, Kanke, Ranchi, Jharkhand, India; 2 Department of Veterinary Biochemistry, Ranchi Veterinary College, Birsa Agricultural University, Kanke, Ranchi, Jharkhand, India; 3 Department of Animal Nutrition, Ranchi Veterinary College, Birsa Agricultural University, Kanke, Ranchi, Jharkhand, India; 4 Department of Veterinary Physiology, Veterinary College, Pookode, Lakkidi, Kerala, India

**Keywords:** body growth, Booroola fecundity gene, Chhotanagpuri sheep, multiple births, single-strand conformational polymorphism

## Abstract

**Aim:**

Chhotanagpuri breed of sheep reared for mutton in Jharkhand, India, having problem of low litter size and body weight. The response of genetic improvement for traits with low heritability through traditional selection method is time-consuming. Therefore, marker-assisted selection based on a polymorphism study of suitable candidate gene can response quickly. Thus, this study was aimed at identification of different allelic and genotypic frequencies of Booroola fecundity (*FecB*) gene and its association with multiple birth and postnatal growth in Chhotanagpuri sheep.

**Materials and Methods:**

DNA isolation and gene-specific amplification of *FecB* gene was performed from blood samples of from 92 Chhotanagpuri lambs maintained under similar feeding and management conditions. Custom nucleotide sequencing and single-strand conformational polymorphism analysis were performed to identify different genotypes with respect to the target gene. Statistical analysis was performed for determination of allelic and genotypic frequencies of *FecB* gene polymorphisms and its association with multiple birth and postnatal growth of lambs from birth to 52 weeks age.

**Results:**

“AA,” “AB,” and “BB” genotypes were found at locus-1 as it is polymorphic for *FecB* gene while locus-2 was found to be monomorphic for *FecB* gene. Higher frequency of “A” allele at locus-1 was found in single born lambs, whereas “B” allele was predominant among multiple born lambs. The lambs having “BB” genotype weighed significantly (p≤0.01) heavier than those of “AB” and “AA” genotype at 52 weeks of age.

**Conclusion:**

“BB” genotype has emerged as favored genotype for multiple births and better growth indicator. Therefore, homozygous lambs for “B” allele should be selected and utilized in breeding program for better growth rate.

## Introduction

Sheep farming is of major economic importance, especially for small and marginal farmers because it requires minimum resource [1]. Inevitably, rearing of Chhotanagpuri sheep in its native tract mainly for mutton purpose is of vast significance in Jharkhand where rural and landless farmers are in the majority due to shortage of arable land. However, sheep production suffers from major constrain as the majority of sheep breeds in India is having low litter size except the Garole, Kendrapara, and NARI-Suvarna sheep breed (http://www.cswri.res.in/breed_profiles.asp).

The prolificacy trait is quantitative in nature and controlled by multiple genes [2]. Improvement of reproductive traits has conventionally been regulated using quantitative genetic methods. Hence, increase in litter size by selection within a breed will be a time-consuming process as the reproductive traits are having low heritability. If the major genes associated with reproduction are identified, they can be introduced in breeding through marker assisted selection and it can infuse superior genotypes rapidly in the breeding population [3,4]. The Booroola fecundity gene (*FecB*) is an autosomal gene, which enhances ovulation rate through codominant effect and litter size by partial dominance [5,6]. The *FecB* locus is situated in the region of ovine chromosome 6, which is syntenic to human chromosome 4 [7]. High prolificacy in Booroola sheep is associated with non-conservative mutation (q249r) in a conserved intracellular kinase signaling domain of the bone morphogenetic protein receptor-1B (BMPR-1B) expressed in ovary and granulosa cells [8,9]. The BMPR-1B, also known as activin-like kinase-6, is a multifunctional protein of transforming growth factor-β superfamily which regulates growth and differentiation in many cell types. In recent years, it has also been shown to be associated with embryogenesis, hematopoiesis, immunological responses, reproductive endocrinology, development of reproductive organs, litter size, and body biometrics [10-13].

Hence, this study has been undertaken to identify different allelic and genotypic frequencies of *FecB* gene and its association with multiple birth and postnatal growth in Chhotanagpuri sheep.

## Materials and Methods

### Ethical approval

All the animal experiments were conducted after approval of committee for the purpose of control and supervision of experiments on animals.

### Experimental design

This investigation was conducted on 92 Chhotanagpuri lambs maintained under Mega sheep seed project, at Instructional Small Ruminant Farm of Ranchi Veterinary College, Birsa Agricultural University, Kanke, Jharkhand, India. The lambs were maintained under natural feeding system during pre-weaning period (0-3 months of age) with their dams. However, adult sheep were maintained under semi-intensive system of management with 7-8 h of grazing daily. To study the growth rate, absolute body weights of Chhotanagpuri sheep were recorded at birth, 4^th^, 8^th^, 12^th^, 24^th^, 36^th^, 48^th^, and 52^nd^ week of age. Birth weights of lambs were recorded within 2 h of birth. Body weights were recorded in the morning before offering feed.

### Collection of blood samples and genomic DNA isolation

The jugular blood samples of 92 individuals (5 ml each) from Chhotanagpuri lambs were collected in vacutainer tubes containing ethylenediaminetetraacetic acid (EDTA) as anticoagulant. Cold chain was maintained throughout the sampling process. Genomic DNA was isolated from white blood cells using standard phenol-chloroform-Isoamyl alcohol method as per the standard protocol described by Sambrook *et al*. [14]. The extracted DNA samples were assessed for quantity and purity using NanoDrop Spectrophotometer (ND-1000 NanoDrop Spectrophotometer, Thermo Scientific, USA) while visual confirmation of the DNA integrity was also assessed by running on 0.6% agarose gel.

### Amplification of *FecB* gene

The 190 bp fragment of the *FecB* gene was amplified from the genomic DNA of Chhotanagpuri lambs by polymerase chain reaction (PCR) using forward (5’- CCAGAGGACAATAGCAAAGCAAA-3’) and reverse primer (5’- CAAGATGTTTTCATGCCTCATCAACAGGTC-3’) reported elsewhere [9]. The amplification was performed in a thermocycler (GeneAmp^®^ PCR system 9700, Applied Biosystems, USA) using PCR reaction mixture containing 1.5 µl 10× PCR buffer, 0.5 µl magnesium chloride (25 mM), 0.5 µl (10 mM) dNTPs, 0.5 µl (20 ng/µl) of each primer, 1U of Taq polymerase (SIGMA, USA), and 1.5 µl diluted genomic DNA (50 µg/µl). The final volume was adjusted to 25 µl by adding nuclease-free water. The thermocycling steps consisted of initial denaturation at 94°C for 3 min; while the cycling parameter consisted of denaturing step of 30s at 94°C, an annealing step of 30s at 58°C and an extension step of 30s at 72°C for 33 cycles; while the final extension step of 7 min was performed at 72°C. A non-template control reaction was simultaneously run to eliminate reagent contamination. The amplified products were resolved on 2% agarose gel in Tris-acetate EDTA buffer (×1). The agarose gel was stained with ethidium bromide and documented under UV light in a gel documentation system (UMAX PowerLook 2100XL-USB with MagicScan, Alpha Innotech Corporation, USA).

Single-strand conformational polymorphism (SSCP) of PCR amplified *FecB* gene fragment.

The SSCP analysis of *FecB* gene was carried out by diluting 4 µl amplified PCR product to 10 µl of SSCP gel loading dye (0.05% bromophenol blue, 0.05% xylene cyanol, 95% formamide, 20 mM EDTA). After denaturation at 95°C for 5 min, the samples were snap chilled immediately on ice for 10 min. Then, the contents were separated using 12% vertical acrylamide: bis-acrylamide gel electrophoresis at 8 V/cm for 16 h under refrigerated condition (29:1 acrylamide to bisacrylamide). The SSCP patterns of DNA fragments were visualized by silver staining of the polyacrylamide gel after electrophoresis [15].

### Purification of PCR products and custom nucleotide sequencing

The PCR products were purified using PureLink^®^ PCR purification kit (Thermo Scientific, USA) to remove primer dimers and other PCR ingredients before custom sequencing following manufacturer’s instructions. Custom sequencing of the purified PCR products from four selected animals was performed in both the directions using DNA sequencing service provided by Xcelris, Hyderabad following BigDye terminator v3.1 Cycle Sequencing Method (Applied Biosystems, USA).

### In-silico analysis of *FecB* nucleotide sequence

The sequence chromatogram was annotated with BioEdit Sequence Alignment Editor Software version 7.0.5 (Isis Therapeutics, USA). The annotated sequences were identified based on being the closest match to the sequences submitted in the nonredundant GenBank nucleotide database (http://www.ncbi.nlm.nih.gov) on BLASTN searches [16].

### Statistical analysis

The genotypic frequency, as well as the frequency of different alleles of *FecB* gene, was computed after sequence alignment. *Chi-square* test was used for statistical analysis in GraphPad Software (GraphPad Software, Inc., USA). The statistical significance of various fixed effect was studied by F-test. Whenever, the effect was significant, the differences were tested for significance by Duncan’s multiple range test as modified by Kramer [17] at 5% level of significance using the inverse of coefficient matrix.

## Results

### PCR amplification of *FecB* gene fragment

**Figure-1:**
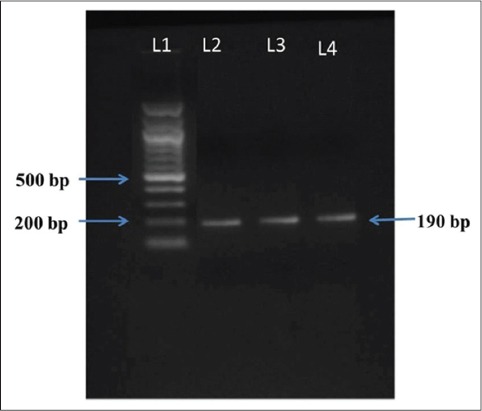
Polymerase chain reaction amplified product of Booroola fecundity (*FecB*) gene (190 bp) along with 100 bp DNA ladder; L1: DNA ladder; L2, L3, and L4: *FecB* gene product.

The 260/280 nm absorbance ratio for all the DNA samples ranged from 1.7 to 1.9 indicating acceptable quality of genomic DNA which was confirmed by visualization in agarose gel. PCR amplified product showed a prominent band of 190 bp size shown in Figure-1.

### SSCP analysis of PCR amplified *FecB* gene product

**Figure-2:**
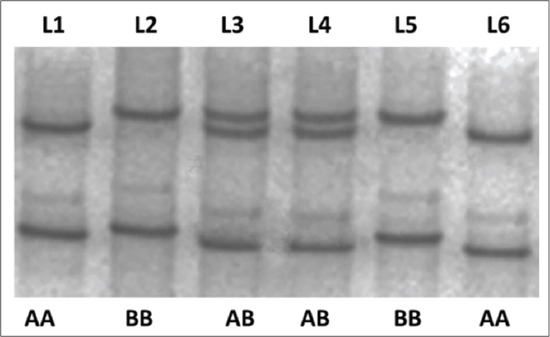
Different genotypic pattern (AA, AB, BB) of Booroola fecundity gene in single-strand conformational polymorphism analysis. L1: AA; L2: BB; L3: BBCD; L4: AB; L5: BB; L6: AA.

SSCP revealed a polymorphic pattern with three genotypes, depicted as AA, AB, and BB, based on different band pattern observed. Three bands were present in the case of AA and BB genotype, whereas four bands were present in AB genotype (Figure-2).

### Nucleotide sequence analysis of PCR-SSCP variants

Manual annotation of the sequence chromatograms by BioEdit Sequence Alignment Editor Software version 7.0.5 revealed gene sequences of the partially amplified *FecB* gene fragment of Chhotanagpuri sheep and submitted to NCBI GenBank (Accession number KX896751). Custom nucleotide sequencing revealed single A/G polymorphism at 746 position (locus-1) of the coding region of *FecB* (BMPR-IB) gene whereas locus-2 was found to be monomorphic at 750 position as compare to reference sequences (AF312016, GU979816).

### Genotype and gene frequency analysis

The genotypic and allelic frequencies in single and multiple born lambs were given in Table-1. The frequency of “A” allele at locus-1 was found to be higher in the case of single born lambs, whereas the frequency of “B” allele was found to be higher in the case of multiple born lambs.

**Table 1:** The genotypic (“AA,” “AB,” and “BB” at locus 1) and allelic (“A” and “B”) frequencies of lambs born in singlet and multiple birth condition on the basis of SSCP variants of *FecB* gene.

Genotypes	Genotype frequency	Allele	Allelic frequency
Singlet condition			
Locus-1			
AA	0.424	A	0.587
AB	0.326	B	0.413
BB	0.250		
Multiple birth			
Locus-1			
AA	0.048	A	0.22
AB	0.343	B	0.78
BB	0.608		

SSCP: Single-strand conformational polymorphism, *FecB*: Booroola fecundity

### Impact of *FecB* gene polymorphism on growth of lambs

Body weights of lambs were studied from birth to 12^th^ week of age at an interval of 4 weeks and thereafter at 24, 36, 48, and 52 weeks of age. Least square analysis of variance was performed to see the effect of *FecB* genotypes (“AA,” “AB”, and “BB” at locus-1)) on body weight of lambs at birth and different stages of growth (Table-2).

**Table 2:** Comparative analysis of body weights (least-square means±SE) with different genotypes (“AA,” “AB,” and “BB”) at locus 1 of *FecB* gene in Chhotanagpuri lambs from birth to 52 week of age.

Effects	Number of observed	Body weights (kg) at different stages of growth							

		Birth	4-week	8-week	12-week	24-week	36-week	48-week	52-week
Overall (µ)	(92)	1.76±0.03	4.04±0.11	5.64±0.18	6.64±0.23	8.42±0.26	9.79±0.28	10.92±0.27	11.83±0.32
Genotypes at Locus 1									
AA	(39)	1.72±0.04	4.10±0.16	5.78±0.26^ab^	6.75±0.33^ab^	8.52±0.38^ab^	9.61±0.39^ab^	10.80±0.38^ab^	11.72±0.45^b^
AB	(30)	1.78±0.04	3.90±0.16	5.33±0.26^b^	6.18±0.33^b^	7.90±0.38^b^	9.26±0.39^b^	10.37±0.38^b^	11.22±0.45^b^
BB	(23)	1.77±0.06	4.12±0.17	5.80±0.27^a^	6.98±0.34^a^	8.85±0.40^a^	10.48±0.42^a^	11.60±0.40^a^	12.54±0.48^a^

a,b,c Values bearing same superscript in a column for each effect separately did not differ significantly (p≤0.01). SE=Standard error, *FecB*=Booroola fecundity

The lambs having “BB” genotype weighed significantly (p≤0.01) heavier than lambs of “AB” genotype from 8 to 52 weeks of age. However, the difference between “AA” and “BB” genotype was found to be non-significant (p≥0.01). Similarly, the difference between “AB” and “AA” genotypes was also found to be non-significant (p≥0.01) up to 48 weeks of age (Table-2). However, finally at 52 weeks of age, the weight of lambs having “BB” genotype weighed significantly (p≤0.01) higher than lambs of both “AA” and “AB” genotype.

### Discussion

Chhotanagpuri sheep as the sole recognized sheep breed of Jharkhand carries ample significance but suffers from low litter size. The *FecB* and related fecundity associated genes such as *FecB, FecX^1^, FecX^H^, FecX^B^*, and *FecX^G^* genes are well recognized to be associated with fertility, growth, and developmental parameters [18-25]. In this study, we have analyzed the frequencies of different genotypes and alleles in different loci of partially amplified 190 bp *FecB* gene fragment and its association with postnatal growth rate up to 52 weeks of age in Chhotanagpuri sheep. Three SSCP variants of *FecB* gene at locus-1 (“AA,” “AB” and “BB” genotypes) were observed. Genotypic frequencies at locus-1 among multiple birth lambs were 0.048, 0.343, and 0.608 for “AA,” “AB,” and “BB” genotypes, respectively, which revealed that “BB” genotypes are favored genotype for multiple births. This is in accordance with the finding of Guan *et al*. [12] where mean litter size of Chinese Merino sheep with genotypes “BB” and “AB” was found to be significantly higher than that of genotype “AA.” Genotypic frequencies at locus-1 revealed that the overall frequency of “A” allele is more (0.587) as compared to “B” allele (0.413). Among multiple born lambs, the frequencies of “B” allele (0.78) was found to be predominant than its counterparts “A” allele (0.22). The present findings are in agreement with the finding of Jia *et al*. [26], who found three types of genotype of *FecB* gene in small tailed Han sheep and poll Dorset sheep. Similarly, Guan *et al*. [12] have also reported three different Booroola genotypes in Chinese Merino sheep. Polley *et al*. [27] reported *FecB* gene polymorphism in Garole sheep and obtained two types of allele of that gene as observed in this study. In a related study by Sun *et al*. [28], on microsatellite markers have revealed different polymorphisms in *FecB* gene having a significant effect on litter size in Hu sheep. Similar studies on related fecundity genes have also documented several polymorphisms, and many of them were found to be associated with fertility parameters in sheep as well as goats. Genome-wide association studies by Demars *et al*. [29] have identified two novel mutations in the BMP15 or *FecX* gene associated with increased litter size and ovulation rate in Grivette and Olkuska ewes. In a related study by Feng *et al*. [30] have revealed GDF9 gene or *FecG* polymorphism is having a positive significant association with high litter size in Jining Gray goats.

However, Chu *et al*. [31] reported that the *FecB* gene had no significant effect on prolificacy of Jining Gray goats whereas Ahlawat *et al*. [32] have shown that *FecB*, *BMP15, and GDF9* genes have no effect on prolificacy of seven Indian goat breeds including the highly prolific Black Bengal goats. Findings of Ghaffari *et al*. [33] in Shal sheep and Borni *et al*. [34] in North African Barbarine ewes suggested that the genetic factor controlling twinning in these sheep breeds is not related to *FecB* mutation. Shafieiyan *et al*. [35] have also reported no mutations of *FecB* and *FecG^H^* genes in high prolific Iranian Lory sheep. The difference in opinion may be due to environmental factors, the difference in breeds or genetic materials used.

This study revealed a significant effect of genotypes at locus-1 on body weights of lambs from 8^th^ to 52^nd^ week of age, though its effect was not significant for birth weight and weight at 4^th^ week of age. At all stages of growth from 8 to 52-week of age, lambs with “BB” genotype weighed significantly more than heterozygous (“AB”) lambs, but the difference with those with “AA” genotype was not significant except at 52 weeks of age. The superiority of “BB” genotype over “AB” for body weights from 8 to 52-week of age ranged between 8.82% (8 weeks) and 13.17% (36 weeks). However, its superiority over “AA” genotype ranged between 0.35% (8 weeks) and 9.05% (36 weeks). This is in accordance with findings of Guan *et al*. [12] where the body weights of “BB/BA” Chinese Merino lambs at 90 days after birth were found to be significantly higher than “AA” lambs. It is also evident that “BB” genotype has emerged as superior to “AA” and “AB” genotypes with the progression of age.

## Conclusion

Lambs of BB genotype were found to be favorable for multiple birth and B allele was found to have better growth potential. Thus, the lambs for “BB” genotype may be selected and utilized for breed improvement under breeding program.

## Authors’ Contributions

TO, LBS, and DKS have designed the research plan TO have performed the wet lab analysis MG, SSK, and RK have analyzed the data. TO, MG, and DKS have drafted the manuscript. All authors have read and approved the final manuscript.
